# Influence of selected factors on the Firmicutes, Bacteroidetes phyla and the *Lactobacillaceae* family in the digestive tract of sheep

**DOI:** 10.1038/s41598-021-03207-w

**Published:** 2021-12-10

**Authors:** Paulina Cholewińska, Magdalena Wołoszyńska, Marta Michalak, Katarzyna Czyż, Witold Rant, Jakub Smoliński, Anna Wyrostek, Konrad Wojnarowski

**Affiliations:** 1 Institute of Animal Breeding, Wroclaw University of Environmental and Life Sciences, 51-630 Wroclaw, Poland; 2 Department of Genetics, Wroclaw University of Environmental and Life Sciences, 51-630 Wroclaw, Poland; 3 Department of Animal Nutrition and Feed Management, Wroclaw University of Environmental and Life Sciences, 51-630 Wroclaw, Poland; 4 Institute of Animal Breeding, Warsaw University of Life Sciences – SGGW, 02-786 Warsaw, Poland

**Keywords:** Genetics, Agricultural genetics, Microbiology, Applied microbiology, Bacteria, Microbial communities, Environmental microbiology

## Abstract

In this study, we used 10 healthy sheep, which gave birth to healthy twins. Stool samples were collected from mothers and their offspring 3 times during the study (0, 28 and 56 day postpartum). Milk samples were taken from the mothers at the same time. RT PCR analysis of faeces and milk was performed in order to assess the level of bacteria from the Firmicutes and Bacteroidetes phyla including the family *Lactobacillaceae* (phylum Firmicutes). The composition of mother's milk was also analyzed and their BCS. The data were compiled statistically. The obtained results showed that the level of the studied groups of bacteria may change due to the change of diet. Additionally, there were significant differences between lambs and mothers in the levels of the studied groups of bacteria. Analysis also shown that in the digestive system of mothers was a smaller disproportion in the level of the studied bacterial phyla than in lambs. The results also indicated the occurrence of differences in the bacterial composition at the individual level, both in ewes and their offspring. Additionally, in the conducted experiment, there were differences in the level of Firmicutes and Bacteroidetes groups depending on the sex.

## Introduction

It is estimated that currently around 3.9 billion ruminants are kept using sustainable agricultural practices, allowing, among other things, the use of undeveloped land through grazing, the use of industrial by-products as a source of food, and the production of energy from low-quality forage with high quality products—milk and meat^1–3^. Ruminants are characterized by a high level of complexity in the digestive system and the microbiome that inhabits it, i.e. the collection of genomes that make up the microbiota. Its role is mainly to process plant particles and convert them into energy for the animal. The substances produced in these processes are volatile fatty acids (VFA—especially glucogenic VFA—the propionic fatty acid), which are the main source of energy (accounting for about 70% of the demand) and have a direct impact on the animal's physiological parameters, such as development, health, production indicators^3–5^. The formation of VFA is possible due to the administration of carbohydrate feed, where VFA are the products of the final fermentation of sugars by bacteria. However, fats are also used to create VFA. Bacteria such as *Anaerovibrio lipolytica* break down fats into glycerol and fatty acids, where the fatty acids are then used to convert proteins in the rumen. Therefore, the appropriate content of both carbohydrates and fats in the feed is important in maintaining the microbiological homeostasis of the ruminant digestive system^6–10^. The bacterial flora of the digestive system of ruminants, also affects the quality of animal products, which has been revealed in many studies, including Tanca’s et al.^6^ and O’Hara’s et al.^7^.

Not only the number of microorganisms, but also their diversity have a significant impact on the animal, especially its health, production indicators and proper development of young animals^8–11^. The most numerous group of microorganisms in the digestive system of ruminants are bacteria. They account for about 10^9^–10^10^/ml of rumen content, in the large intestine there are 400 species, i.e. 10^10^–10^12^/g of colony forming units (CFU). The existence of individual species and their number depend on factors such as diet, environment, health and heredity^12,13^. Phyla Firmicutes and Bacteroidetes are the most abundant in all ruminants, Proteobacteria and Fibrobacter are less frequently observed, while Tenericutes and Actinobacteria occur in small amounts^10^, which is related to the diet of ruminants, i.e. plant food. The most numerous bacterial phyla are associated primarily with food with a large amount of fiber and polysaccharides^14,15^. The main role of bacteria in the ruminant's digestive tract is the decomposition of cellulose and hemicelluloses^16^, but they are also involved in the digestion of other nutrients such as fats and proteins^16,17^.

The aim of the study was to assess the level of selected phyla and families of bacteria in the digestive system of sheep, taking into account changes related to nutrition, growth and development of lambs, individual variability and sex.

## Results

### BCS condition

**Figure 1:**
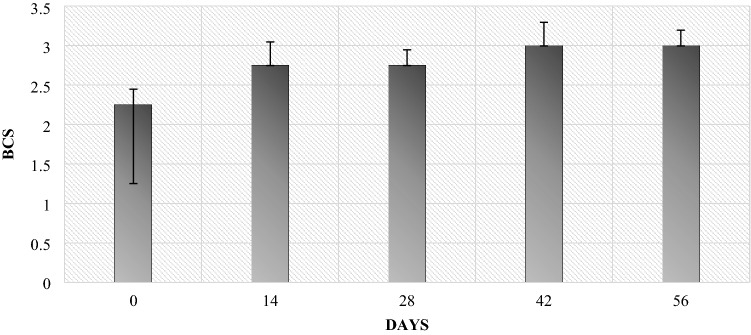
The condition of ewes from the day of birth to 56 days after lambing (no significant differences between periods were found).

The results of the condition assessment carried out by the BCS method of ewes showed that the lowest fitness occurred on the day of lambing (day 0)—2.25. On the other hand, on the 14th and 28th days after lambing, it improved and increased to 2.75. On the postpartum day 42, there was a further increase in BCS to a level of 3, which was maintained for the next two weeks of the experiment, until day 56 (Fig. 1).

**Figure 2:**
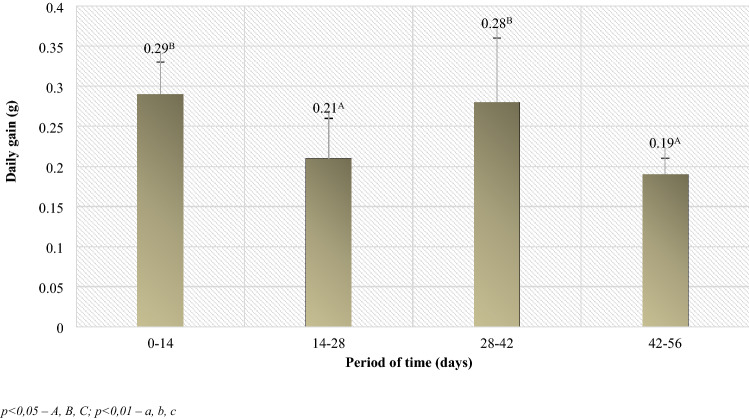
Average daily gain of lambs between the control weighing periods (kg). *p* < 0.05—A, B, C; *p* < 0.01—a, b, c.

The authors reported that BCS condition of lambs and mothers was the lowest on the day of lambing. However, along with the stabilization of the postpartum situation, the condition improved. On days 28 and 42, they reached the normal level (close to 3.0). However, on day 56 there was a slight decrease, which could have been related to the beginning of the grazing period on day 42 after giving birth (Table 1). On the other hand, when analyzing the development of body weight, it was found that during the first 14 days of rearing, the body weight of the studied lambs increased by 4.07 kg, and during the next 14 days (days 14–28 of rearing), the weight gain was 2.93 kg (Table 2). The differences in the development of body weight between the above mentioned periods reflect the estimated daily gains in lambs (Fig. 2), where the growth rate over the period of 0–14 days was statistically significantly higher than in the daily gains in lambs between 14 and 28 days of age. On the other hand, the body weight obtained by the lambs on the 42nd day of rearing indicated a renewed improvement in their growth rate in the period of 28–42 days, which is indicated by statistically significantly higher daily gains compared to the previous period (Fig. 2). Another statistically significant reduction in the rate of weight development of lambs took place between 42 and 56 days of age, when there was a change in diet related to their going to the pasture and the cessation of beet pulp and lupine feeding. Generally, however, it can be stated that the growth rate of the lambs in the rearing period up to the 56th day of life was satisfactory. The daily gains achieved by the lambs at that time exceeded 200 g/day (Table 2).

**Table 1:** Lambs body weight and BCS assessment.

Day 0	*SD*	Day 14	*SD*	Day 28	*SD*	Day 42	*SD*	Day 56	*SD*
Lambs body weight (kg)									
3.2	1.2	7.27	1.5	10.2	2.0	13.67	2.8	16.33	3.2
BCS assessment of lambs									
2.75	0.4	2.8	0.2	2.8	0.4	3.0	0.4	2.8	0.2

**Table 2:** Daily mass gains in lambs during the rearing period up to 56 days of age (kg).

Time period (days)	Daily gains (kg)	*SD*	p-value	Time period (days)	Daily gains (kg)	*SD*	p-value
0–28	0.25^A^	0.02	0.02	14–42	0.23	0.01	0.06
0–42	0.25^A^	0.01	0.02	14–56	0.21^B^	0.02	0.02
0–56	0.23	0.01	0.34	28–56	0.22	0.01	0.07

*p* < 0.05—A, B, C; *p* < 0.01—a, b, c.

### Milk analysis

**Figure 3:**
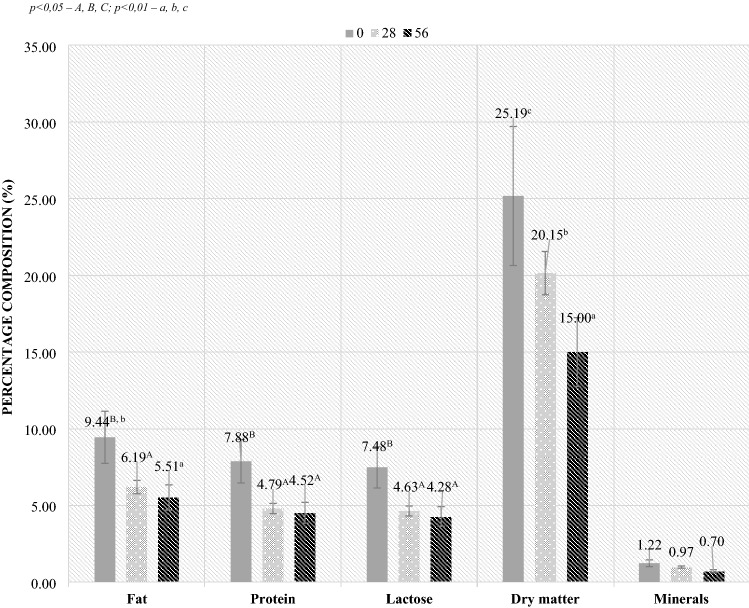
Composition of ewe’s milk depending on the collection day (day 0, day 28, day 56). *p* < 0.05—A, B, C; *p* < 0.01—a, b, c.

In the case of the analysis of the milk, the highest fat content was found on the day of lambing, while with the progressive lactation and the period of growth of the lambs, the level of fat in milk decreased significantly. The largest differences in the composition of milk occurred in relation to the content of protein, lactose, dry matter and minerals. Their highest level was observed during lambing (day 0), while later (from day 28 to day 56 of lactation) all studied parameters have decreased significantly (Fig. 3).

**Figure 4:**
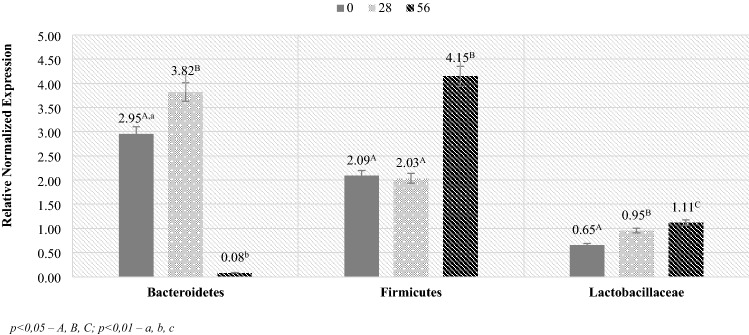
RNE (relative normalized expression) level in the ewes milk. *p* < 0.05—A, B, C; *p* < 0.01—a, b, c.

The next step in the analysis was to determine the level of selected phyla and families of bacteria in milk (Fig. 4). In the case of the analysis of milk samples, significant differences in the level of RNE values in the Bacteroidetes phylum were found. The highest value was recorded for milk on day 28, and the lowest value for milk taken on day 56 post lambing. There were no statistically significant differences between days 0 and 28. For the Firmicutes phylum and the *Lactobacillaceae* family, no significant differences were found between the RNE values, although an upward trend in RNE values was observed for the Firmicutes phylum between days 28 and 56 (doubling the value).

### Real time results of feces

**Figure 5:**
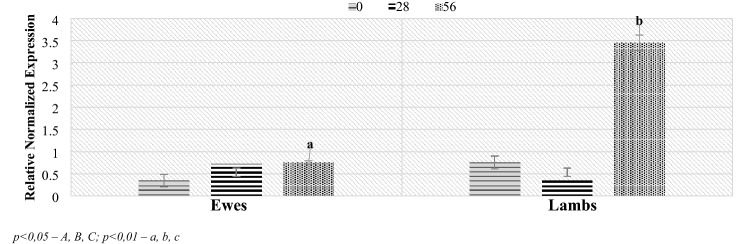
Level of Bacteroidetes (RNE) in ewes and lambs feces. *p* < 0.05—A, B, C; *p* < 0.01—a, b, c.

**Figure 6:**
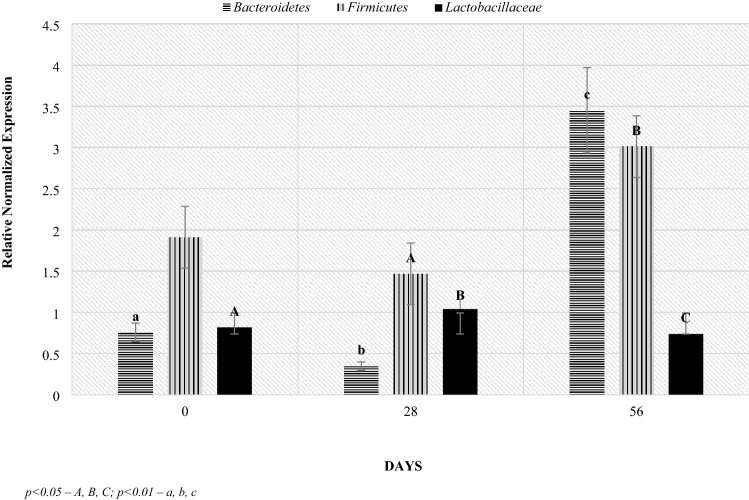
Level of RNE in lamb feces. *p* < 0.05—A, B, C; *p* < 0.01—a, b, c.

**Figure 7:**
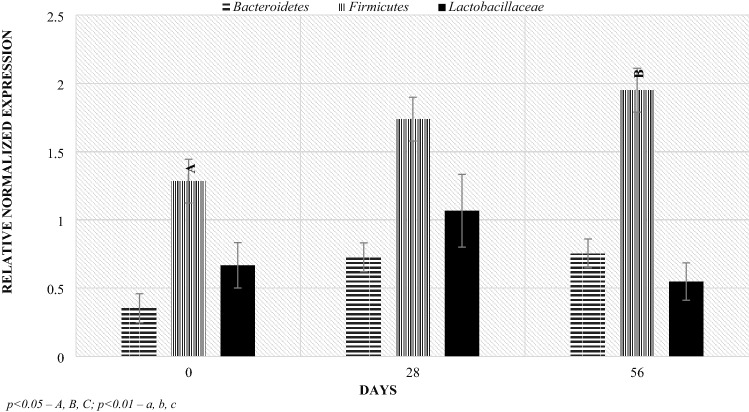
Level of RNE in ewes feces. *p* < 0.05—A, B, C; *p* < 0.01—a, b, c.

**Figure 8:**
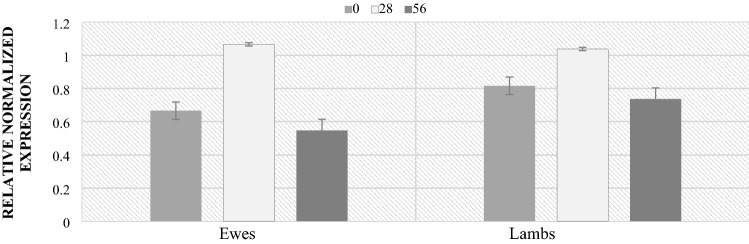
Level of *Lactobacillaceae* (RNE) in ewes and lamb feces.

Real-time PCR analysis in relation to the level of bacterial groups (Figs. 5, 6, 7, 8): Firmicutes, Bacteroidetes and the *Lactobacillaceae* family allowed to determine the Relative Normalized Expression (RNE) value in relation to the reference gene. Depending on the age of the lambs, in the Bacteroidetes phylum, significant differences were found in the feces of the lambs between the day of lambing and 56 days after lambing and between days 28 and 56 after lambing. The initial RNE value was around 0.76, then it decreased to around 0.36, after which the value increased tenfold. Additionally, on day 56 after lambing, significant differences in the level of this index were shown between mothers and lambs (Fig. 5).

A higher level of Bacteroidetes phylum was observed on the 56th day postpartum in lambs (about 3.45) than in mothers (about 0.75). On day 28 postpartum, the RNE level in lambs was half lower than in the mothers, while the opposite occurred on day 0. Additionally, statistically significant differences were found between the day of birth and the 28th day of life (p = 0.031) and between 28 and 56 day of lambing (p = 0.0098) in the RNE level in the case of lambs, which was not observed in the mothers. At 56 days of age, the level of the Bacteroidetes phylum in stool samples increased significantly, while from the day of lambing to the 28th day after lambing, its level decreased.

The RNE levels for the Firmicutes phylum remained similar in lambs and dams until day 28 post lambing, although on day 0 there was an upward trend in lambs compared to dams. On the other hand, the RNE level at day 56 postpartum was approximately one unit higher in lambs (3.012 and 1.951, respectively). Regarding the lactation period, significant maternal differences were observed between days 0 and 56 postpartum where the value was approximately 0.7 higher. On the other hand, in the case of lambs, significant differences were shown between days 28 and 56 after parturition, where the effect of diet on RNE levels was shown (where the RNE levels could be affected by a change in the diet associated with going out to pasture on day 42 of lambing), which was not observed in mothers.

In the case of the *Lactobacillaceae* family, significant differences in the level of RNE were found between the day of lambing and 28, between 28 and 56 and between 0 and 56. However, no differences were found between lambs and their mothers in the subsequent stages of the study.

**Figure 9:**
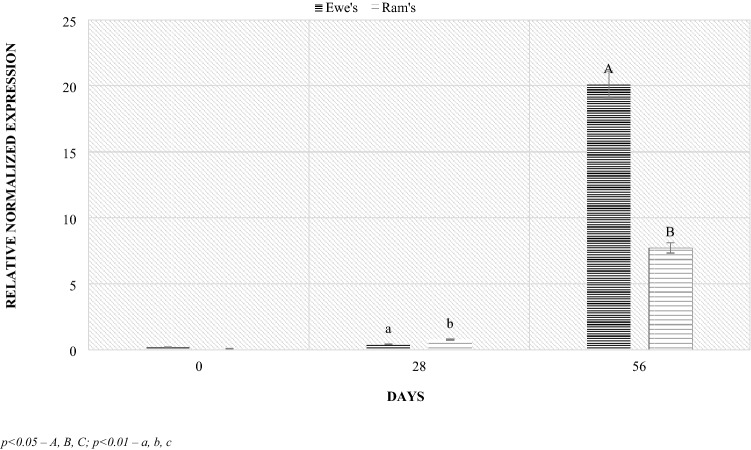
Level of *Bacteroidetes* (RNE) depending on sex. *p* < 0.05—A, B, C; *p* < 0.01—a, b, c.

**Figure 10:**
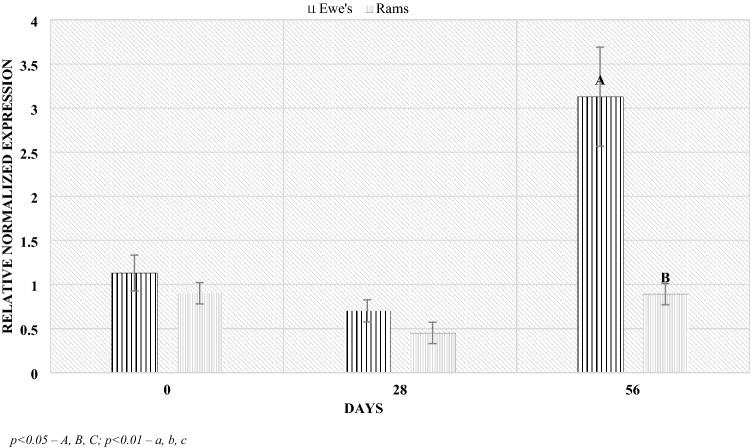
Level of *Firmicutes* (RNE) depending on sex. *p* < 0.05—A, B, C; *p* < 0.01—a, b, c.

**Figure 11:**
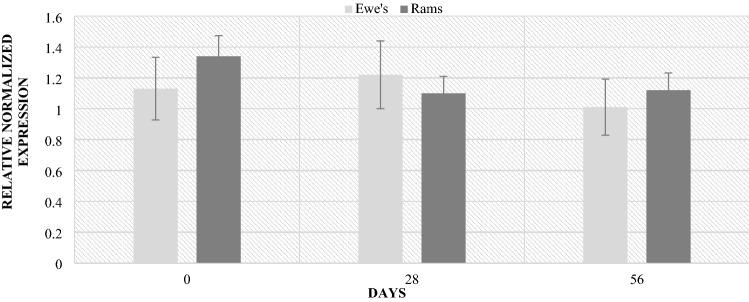
Level of *Lactobacillaceae* (RNE) depending on sex.

The analyzes of lambs feces also showed differences in microbiological composition depending on sex. The ewes to lambs ratio in the study group was 9:11. The highest levels of the Bacteroidetes phylum occurred regardless of sex in 56 postpartum. Significant differences in the phylum level by sex were demonstrated during this period. The ewes were characterized by higher level compared to the lambs (Fig. 9). Similar relationships, also on day 56 postpartum, were found in the level of the Firmicutes phylum. The ewes were characterized by a significantly higher (4 times) level of the phylum compared to the lambs (Fig. 10). However, in the case of the *Lactobacillaceae* family, no significant differences in level depending on sex were found (Fig. 11). However, at lambing and 56 days of age, there was a slightly higher *Lactobacillaceae* family level in lambs feces, while the opposite trend was observed at 28 days of age.

**Figure 12:**
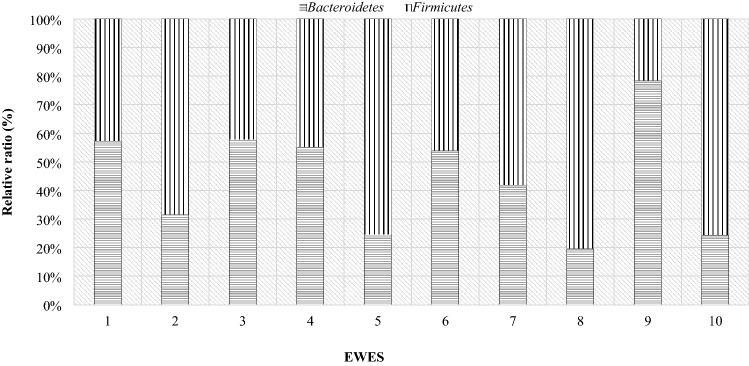
Relative ratio (%) of *Firmicutes* and *Bacteroidetes* phylum on the day 0 in ewes feces.

In order to present individual differences in terms of the microbiome composition, comparisons were made of the occurrence of particular bacterial phyla in the studied individuals. The results show the difference between the individual animals in both dams and lambs. Half of the mothers had higher levels of Bacteroidetes than Firmicutes on the day of lambing, while the other half had the opposite ratio (Fig. 12). The highest level of the Firmicutes phylum occurred in mothers 2, 5, 8 and 10. However, the highest level of Bacteroidetes in mother number 9, and in mothers number 1, 3, 4 and 6, the level was higher, but with similar values (in the range of 50–60%).

**Figure 13:**
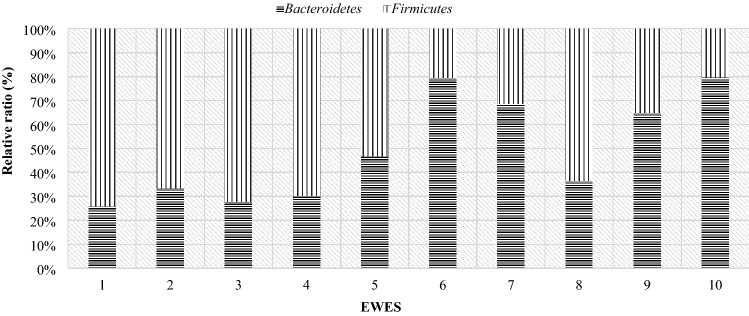
Relative ratio (%) of Firmicutes and Bacteroidetes phylum on the day 28th in ewes feces.

On day 28 postpartum, more than half of the mothers had a higher level of Frimicutes than Bacteroidetes (Fig. 13), although in ewes 6, 7, 9 and 10 the level of the Bacteroidetes was almost 2 times higher than that of Firmicutes. Compared to the day of lambing, some mothers had an increase in Firmicutes and a decrease in Bacteroidetes. In comparison to the day of lambing, fecal samples from maternal numbers 5 to 8 and 10 showed an increase in the level of the Bacteroidetes phylum. In contrast, in ewes numbered 1, 3, 4, and 9 there was a decrease in the level of this phylum. Mother number 2, on the other hand, had a similar level of both phylum’s.

**Figure 14:**
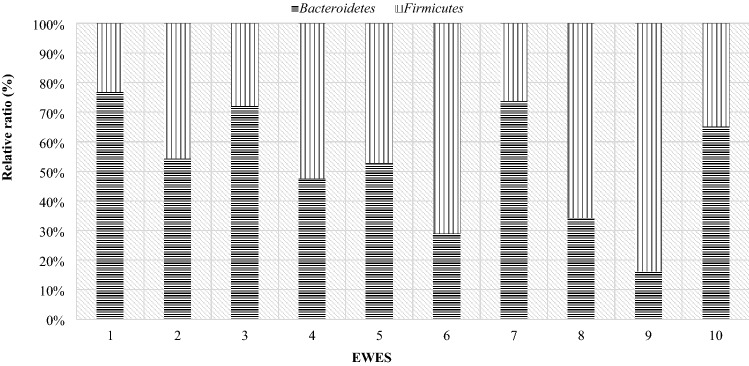
Relative ratio (%) of Firmicutes and Bacteroidetes phylum on the day 56th in ewes feces.

At day 56 postpartum, most of the mothers’ digestive systems were dominated by the phylum Bacteroidetes (Fig. 14). Six mothers had an increase in the Bacteroidetes phylum and a decrease in Firmicutes levels compared to day 28 postpartum. In the case of dams 6, 9 and 10 there was an increase in the share of Firmicutes and a decrease in Bacteroidetes. Mother number 8 had similar levels in both phyla compared to day 28 postpartum.

**Figure 15:**
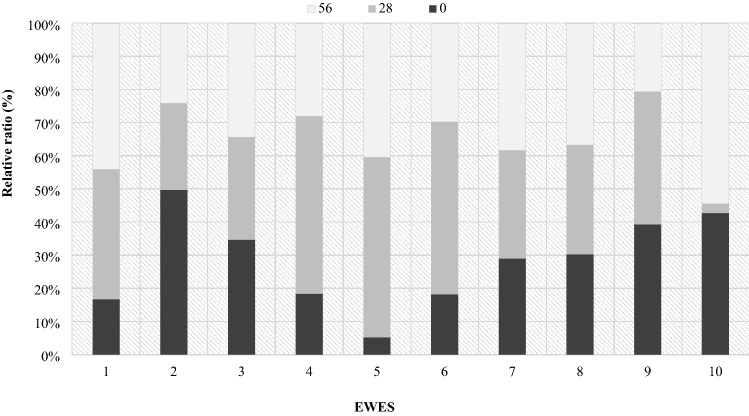
Relative ratio (%) of *Lactobacillaceae* in ewes feces depending on days.

The analysis of the *Lactobacillaceae* family showed significant individual differences (Fig. 15). RNE values were similar in mothers number 7 and 8. However, in the case of the rest of mothers, the share of this family varied considerably in terms of individuals, which was presented in Fig. 15. Individual differentiation in the occurrence of individual bacterial phyla in the period from lambing to day 56 of lactation confirm the estimated correlations, which turned out to be low and statistically insignificant.

The correlation between the level of the Firmicutes phylum in the maternal digestive system and the individual components of milk (protein, dry matter, lactose, fat) was also estimated. There was a statistically significant negative correlation between the discussed bacterial phylum and the protein on day 28 after lambing, which proves that the increase in the level of the Firmicutes phylum reduces the amount of protein in milk. Similar results were obtained for the amount of lactose in milk and the level of this phylum. However, on day 56 postpartum, the correlation for lactose was positive, indicating that if one factor was increased, the other did the same. In the case of the Bacteroidetes group, a statistically significant positive correlation occurred in the period from the day of lambing to the 28th day postpartum, in terms of fat content (Table 3).

**Table 3:** Correlation coefficients between the studied groups of bacteria in the digestive system of ewes and the composition of their milk.

	DAY 0	DAY 28	DAY 56
**The level of *Firmicutes* in the mothers digestive tract and the level of selected milk components**			
Protein	0.40	− 0.75*	0.12
Lactose	0.11	− 0.73*	0.65*
Fat	0.09	0.12	0.22
Dry matter	− 0.38	− 0.32	0.11
***Bacteroidetes* level in the mothers digestive tract and the level of selected milk components**			
Protein	− 0.32	− 0.19	0.43
Lactose	− 0.12	0.20	0.29
Fat	0.09	0.54*	0.12
Dry matter	0.18	0.32	0.37

*p < 0.05.

**Figure 16:**
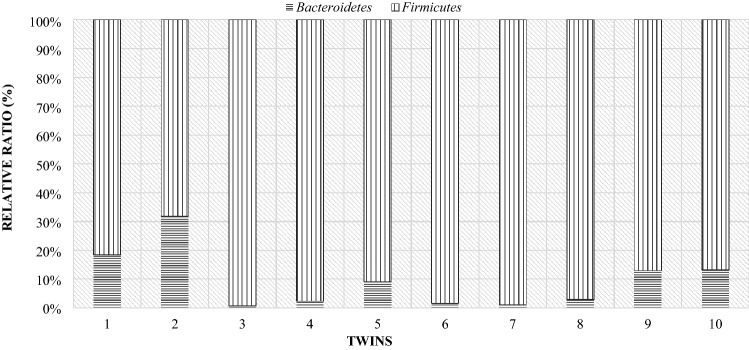
Relative ratio (%) of Firmicutes and Bacteroidetes phylum in lambs digestive tract at day of birth (day 0).

**Figure 17:**
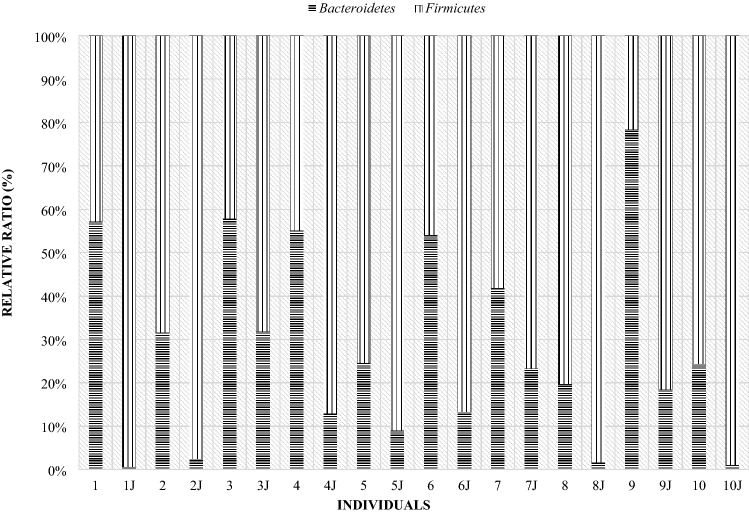
Compare of Firmicutes and Bacteroidetes phylum at day of birth in mother and their twins digestive tract (day 0) (1—mother, 1 J—twins).

As in the case of the mothers, a comparison of the individual differences between the particular phyla of bacteria in lambs was made. Due to the fact that twins from one mother differed slightly in the level of the studied phyla, the comparison was made for individual pairs of twins (Fig. 16). In all pairs of twins on the day of birth, the Firmicutes phylum (over 70%) had a significant share. In the comparison showing the level of this phylum of bacteria in mothers and their twins on the day of lambing, it turned out that the share of the Firmicutes phylum was higher in lambs than in their mothers (Fig. 17).

**Figure 18:**
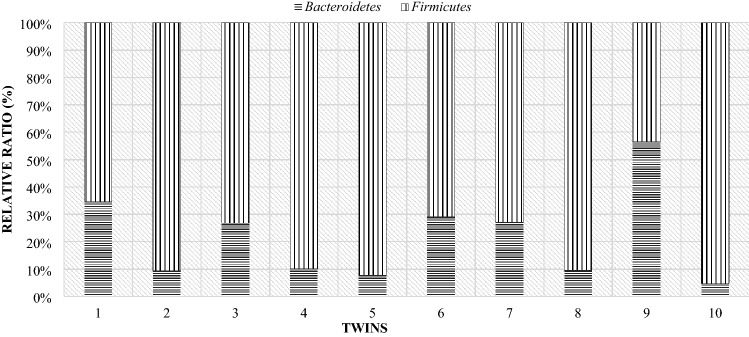
Relative ratio (%) of Firmicutes and Bacteroidetes phylum at 28th day life of lambs.

**Figure 19:**
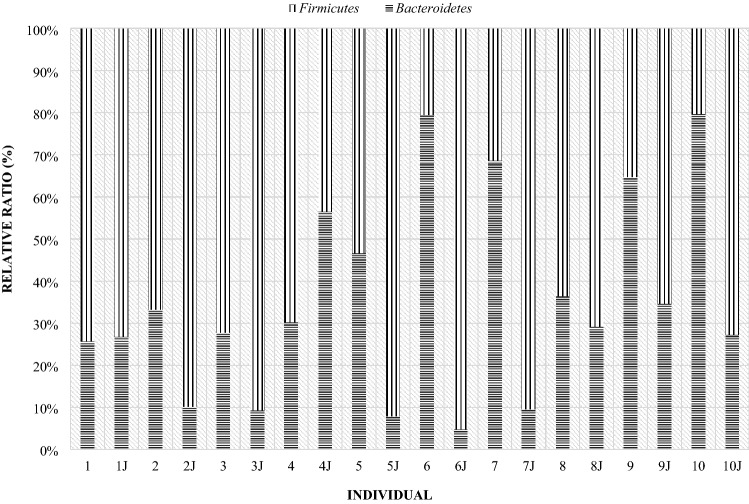
Comparison of Firmicutes and Bacteroidetes phylum at 28th day in mother and their twins digestive tract (1—mother, 1 J—twins).

On the 28th day of twins' lives, as on the day of birth, the Firmicutes phylum was the largest group, with the exception of pair 9 (Fig. 18). For most couples, the results showed that the Bacteroidetes phylum accounted for less than about 35%. Only in the case of the ninth pair, this phylum made up more than half. However, compared to the mothers, the majority of lambs still had lower levels of the Bacteroidetes than the Firmicutes phylum (Fig. 19).

**Figure 20:**
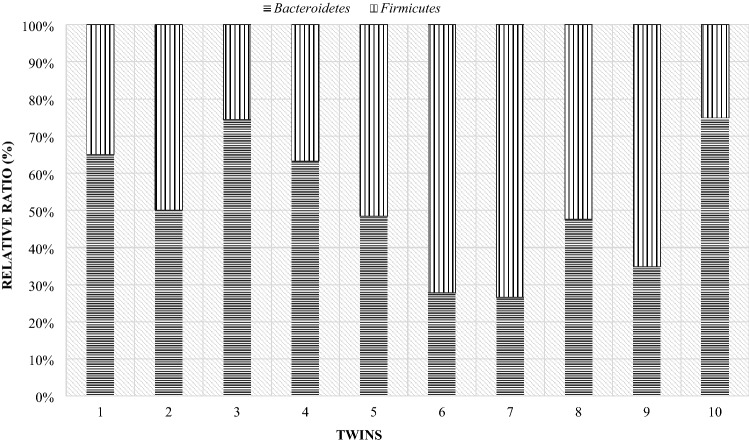
Relative ratio (%) of Firmicutes and Bacteroidetes phylum at 56 days of age in twins.

**Figure 21:**
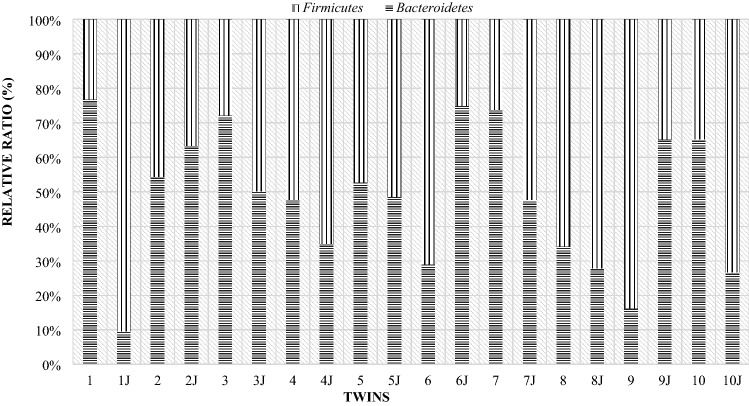
Comparison of the Firmicutes and Bacteroidetes phylum at day 56th postpartum in ewes and their offspring (1—mother, 1 J—twins).

On day 56 postpartum, the lambs increased in the level of the Bacteroidetes phylum relative to Firmicutes compared to day 28 postpartum. In half of the lambs, the Bacteroidetes phylum had a greater share (Fig. 20). The opposite situation occurred in twins with numbers from 5 to 9, in their case the group Firmicutes constituted the larger unit. However, compared to the mothers (Fig. 21), most lambs had lower levels of Bacteroidetes and higher levels of Firmicutes phylum. Estimated correlations in terms of the mutual influence of the Bacteroidetes and Firmicutes in the digestive system of lambs and mothers showed that the mothers had a statistically significant negative correlation in the 24 h postpartum period. On the other hand, in lambs, a statistically significant linear relationship was demonstrated between the studied groups of bacteria during the entire observation period.

**Figure 22:**
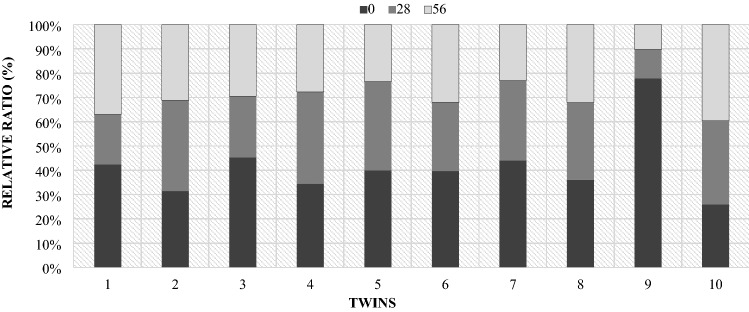
Relative ratio (%) of the *Lactobacillaceae* family in individual pairs of twins depending on the days.

In twins, the levels of the *Lactobacillaceae* family were generally similar compared to the mothers. Still, there were differences between individual pairs of twins, which is presented in Fig. 22. The estimated correlations between the maternal microbiome and the share of individual bacterial phylum’s in their lambs (Table 4), in turn, did not show a statistically significant linear relationship in majority of cases.

**Table 4:** Correlations between the microbiome of mothers and lambs in the studied periods.

Day	0	28	56	The whole period
Firmicutes	0.09	0.05	0.02	0.01
Bacteroidetes	0.24	0.01	− 0.14	0.03
*Lactobacillaceae*	− 0.12	− 0.11	− 0.73*	− 0.38

*p < 0.05.

Estimated correlations between the level of individual groups of bacteria and the digestive system of lambs and components of their mothers' milk showed a statistically significant negative relationship between the cluster Bacteroidetes and the level of lactose and dry matter in milk. On the other hand, when taking into account the entire period of development of the lambs, the fat content was statistically significantly positively correlated with the level of this cluster,. At birth, a negative correlation was found between the level of protein in milk and the *Lactobaciilaceae* family in the digestive system, a positive correlation was shown between the level of lactose and this family in the period from 28 to 56 days of life of lambs, while in the period from the day of birth to 28 days of life in the case of fat (Table 5).

**Table 5:** Correlations between the microbiome of mothers milk/colostrum and lambs in the studied periods.

Day	0	28	56	The whole period
**Correlation between milk/colostrum composition and level of *Bacteroidetes* phyla in lambs digestive tract**				
Protein	0.07	− 0.11	− 0.14	− 0.32
Lactose	− 0.42	− 0.3	− 0.25	− 0.50*
Fat	− 0.18	− 0.40	0.30	0.54*
Dry matter	0.13	0.32	0.45	− 0.50*
**Correlation between milk/colostrum composition and level of *Lactobacillaceae* family in lambs digestive tract**				
Protein	− 0.62*	0.33	− 0.27	0.33
Lactose	0.05	0.36	0.66*	0.42
Fat	0.43	0.54*	0.40	0.45
Dry matter	0.09	0.32	0.21	0.24

*p < 0.05.

## Discussion

During present studies the bacterial composition was determined with regards to two phyla: Bacteroidetes and Firmicutes, which are the most numerous in the digestive system. *The Firmicutes* group includes Gram-positive bacteria, mainly of the *Clostridium* and *Bacilli* classes, which are primarily responsible for the decomposition of exogenous peptides and amino acids. Another phylum—Bacteroidetes is characterized mainly by the ability to decomposition of cellulose, hemicellulose and pectins in the ruminant digestive system. This group includes Gram-negative, anaerobic and relatively aerobic bacteria^15,18–20^.

The current research revealed changes, depending on the developmental stage, in the bacterial composition of lamb feces in relation to studied bacterial groups. Similar results were obtained during studies on cattle conducted by Yáñez-Ruiz et al.^21^ and Li et al.^4^. The lowest levels of, both, *Firmicutes* and *Bacteroidetes* were recorded on the 28th day after birth. The highest level of gene expression of the studied phyla was demonstrated on day 56 after lambing, where the RNE value was the highest. However, at lambing bacteria from both groups were detected, which may confirm the theory that the digestive system is already inhabited by microorganisms in the post-prenatal (from the birth time) period, which has been confirmed by the studies of Yáñez-Ruiz et al.^21^ on calves. It has been shown that as early as between the 1st and the 2nd day after birth, strict anaerobes, that occur in adult animals, are present in the digestive system of young animals. Similar results were also obtained by Wang et al.^14^ where, as in the present research, the majority were *Firmicutes* and *Bacteroidetes*.

The composition of colostrum and milk at first week’s of life also influences the bacterial composition of the digestive system of lambs, which was also shown in the correlation analysis (in case of Bacteroidetes phyla and *Lactobacillaceae* family). During the first weeks of life, the rumen of newborns does not function and the milk passes directly into the abomasum via the intestinal villi. The samples of ewe milk analyzed in the presented study were characterized by the highest level of fat, protein, lactose and minerals on the day of lambing, and their amount gradually decreased during lactation, which is a normal phenomenon^4,22,23^. In addition, the analysis showed that there was a correlation between the composition of milk and the level of bacteria in the digestive system of lambs. The effect of mother's milk on microbes of the young was demonstrated by Addis et al.^24^ and Misztala et al.^23^.

In the period between the 28th and 56th day after parity, a significant increase in the expression level of the Bacteroidetes phylum was noticed, which may indicate an increase in the level of anaerobic bacteria responsible, among others, for the decomposition of cellulose, hemicellulose and pectins. In addition, the level of expression of bacteria from the Firmicutes phylum also increased, while *Lactobacillaceae* showed a downward trend. The decrease in growth rate at 14–28 day of life may have been due to the lambs starting to consume more solid feed. This period is associated with the development of the rumen, which is also a heavy burden for the young organism. On the other hand, the decline in growth rate in the period between 28 – 56 days of age was probably related to the change in diet, which is also described below. Wang et al.^14^ in his studies obtained different results. It may be related to access to the pasture (on day 42 of observation) and a significant change in the composition of the feed and the development of lambs, which was found in the studies by Jami et al.^4^ and Rey et al.^25^.

The influence of the energy level in the ration could have had a key effect on the results, as described in the work of, among others, Lv et al.^26^. Changes that took place in the level of the Firmicutes in lamb faces may also indicate a higher number of anaerobic bacteria, at the expense of a decrease in aerobic activity—mainly lactic acid bacteria. The phenomenon of an increase in the activity of anaerobic bacteria in relation to the decrease in the activity of aerobic bacteria has been described in studies on calves, where it was established that between the 6th and 8th week of the animal's life the number of aerobic and relatively aerobic bacteria decreased and their place was replaced by anaerobic bacteria^8,27^.

These types of changes in lambs and calves are most likely associated with the introduction of solid feed and a reduction of the amount of milk consumed. Additionally, in the present study, at 6 weeks of age, there was a change in the composition of the diet of lambs due to the beginning of the pasture period (pasture was used for about 8 h a day, and was a significant part of the diet). In the case of the Bacteroidetes, diet has been shown to influence the level of this phylum, which was also shown in correlation analysis. The level of Bacteroidetes was possible influenced by the composition of milk. On the other hand, the level of Firmicutes bacteria in the lamb digestive system was mainly influenced by their level in milk. The amount of the *Lactobacillaceae* family in lambs was significantly influenced by milk components, which was also shown in the studies by Tanca et al.^6^, Menzes et al.^28^ and Fernando et al.^29^.

According to the literature data, diet significantly influences the microbiological composition of the digestive system, however, only if it is properly adjusted to animals (in terms of the type of use). Thus, it is possible to stabilize the microbiome in the later period of adaptation to the environment animals are to be kept in^11,26,30^. The commencement of solid feed intake and, consequently, the initiation of rumen fermentation and the development of intestinal villi increases the multiplication of anaerobic bacteria in the digestive system. During this period, these bacteria enter the digestive system, not only through contact with the external environment of the young animal^4,31,32^.

In the presented study, however, lambs showed a significant increase in the expression value of genes from the Firmicutes and Bacteroidetes phyla on day 56 after birth, which resulted in the leveling of RNE values, which were previously differentiated, as in the mothers. The observed even level of expression of the phyla was the result of the beginning of the grazing period, i.e. a change in the diet, and the initiation of an intensive fermentation in the rumen, which resulted in the digestive system not having a well-stabilized microflora^10,13,21,33^.

In the case of the mothers, it was shown that the bacterial composition changed mainly in the level of RNE values of the Firmicutes phylum. The lowest levels occurred during lambing, which could have been a stress factor. However, there was an increase in the RNE level later on, which may suggest a stabilization of the phylum level. On the other hand, in the case of the Bacteroidetes phylum and the family of *Lactobacillaceae*, the lack of changes in the level of expression values may indicate the adaptation of bacteria to the environment in which animals live and to an appropriate current diet. In the case of sudden modification of the diet, there were changes in the bacterial structure of the digestive system, especially in the rumen, which may have led to an increase in the number of unfavorable bacteria^4,34^. In the case studied, bacteria from the Firmicutes phylum constituted a higher level as compared to Bacteroidetes, which is accordant to the results by Wang et al.^14^, Tanaca et al.^6^ and Kim et al.^35^. The values may be related to the diet, because in our case the diet was based mainly on hay with the addition of concentrated feed, during the period of the ewes' stay in the sheepfold, there were no significant changes in the level of the Bacteroidetes phylum in relation to Firmicutes^28,29^. The obtained results of the expression level of the studied phyla are confirmed in the research by Jami et al.^4^ and Rey et al.^25^, wherein similar relationships were shown in the 2 years old cattle—the level of Firmicutes was higher than that of Bacteroidetes.

When comparing maternal and lamb RNE values, there are differences on day 56 after birth in the Bacteroidetes phylum. The level in lambs is 3 times higher, which could be related to the factors described above. Besides, the level of expression value of the studied groups of bacteria is similar. Still, on day 0, there are slightly higher values in the lambs than in the mothers. We observed the opposite situation on the 28th day after birth. The family of *Lactobacillaceae,* on the other hand, maintained a very similar level, showing no upward or downward trend. In the case of *Lactobacillaceae* in the digestive system of lambs, the main factor influencing their amount was the composition of maternal milk, where the level of this bacterial family is relatively constant regardless of the sampling time or diet change. However, the correlations additionally showed an influence of the mother on the level of these bacteria, which may suggest that both milk and the bacterial composition of the vagina along with the oral microbiome (salvia) could have an impact on their levels, which was also suggested in the work of Rey et al.^25^ and Henderson et al.^13^.

Furthermore, in the case of day 0 and 28 days after birth, there was a similar proportion of Firmicutes to Bacteroidetes bacteria in both lambs and ewes (in the case of ewes also on day 56 after birth), as was revealed in the studies on calves and cattle conducted by Jami et al.^4^ and Rey et al.^25^. In this study, the Firmicutes was more numerous than Bacteroidetes. Congruous results were obtained by Douglas et al.^36^ and Zeng et al.^15^. These outcomes may indicate the correct level of both groups in the digestive system of sheep, which can also be confirmed by the condition of the tested animals—correct in terms of age and physiological condition (as revealed by means of indicators expressed by BCS values; within the scope of 2.5–3.0). The obtained results show the existence of interdependencies between bacteria also described by Segata et al.^37^. However, in the case of mothers, the previously mentioned stabilization of the digestive system is visible, which is related to their adaptation to the conditions in which they are kept, as well as to the correct diet and maintenance conditions^13,21^.

Recent studies, including these by Mamun et al.^38^ or Markle et al.^39^ indicate that not only diet and the environment have a significant impact on the microbiological composition of the digestive system of ruminants but also, both, genetic and biological factors. The results of the study suggest that the sex of the lambs could be a biological factor influencing the levels of Firmicutes and Bacteroidetes^38–40^. According to studies by Meon et al.^40^ and Markle et al.^39^ the microbial composition may be influenced by sex hormones and vice versa. Therefore, it can be suspected that the significant differences in the level of phyla between lambs and ewes at 56 days of age were related to the level of sex hormones. However, this phenomenon is not fully understood in the case of ruminants.

Many research on the microbiome suggests that the individual is a significant factor^10,41^. In human studies carried out as part of the Human Microbiome Project^41^, scientists suggest that each individual has its own microbiological composition of the digestive system. Similar conclusions are more and more often described even in ruminants, such as in the studies by Lopes et al.^42^. Our observations indicate that the microbiomes of adults and young individuals are differentiated individually. However, the microbiome of twins is significantly similar in terms of phyla and families by the day 56, which may be due to the fact that, both, individuals in the pair were exposed to the same factors—the maternal microbiome and milk^43,44^. However, variations in the level of phyla or families occurred between pairs of twins, as well as between mothers and mothers and their offspring. In the studies by Mamun et al.^38^ or Zhang et al.^45^, individual differences between studied sheep were shown, confirming the results of this research.

Summary, in the case of lambs there is a visible change in the levels of bacteria, which shows their sensitivity to external factors during the study, such as the composition of the feed. The study also noted the occurrence of individual and statistically relevant gender differences. Interestingly, comparing the results for sheep with cattle, a similarity can be observed in the basic composition of the gastrointestinal microbiome. Therefore, it is important to further investigate the microbiological composition of young ruminants to ensure their proper development. The extensive microflora of the digestive system of ruminants is an indispensable aspect of their health. The study of the composition of their digestive system may allow for the manipulation of the microbiological composition in the future, e.g. by means of diet and/or feed additives in order to prevent animal diseases or increase production efficiency. However, this requires further research.

## Materials and methods

### Animals

The animals used in the experiment were Olkuska sheep, a breed of prolificity at the level of 200% and a good maternal instinct. Rams and ewes are sexually mature at 10 months of age, it is a seasonal breed^46^. The research was conducted on 10 mothers which gave birth to twins—age: 2 (second lambing), weight before pregnant: 48.5 (± 3.25 kg). The main selection criteria were the good health (without symptoms such as diarrhea, increased body temperature, apathy, lack of appetite, etc.) of the mothers and their lambs, and ease of lambing.

### Housing and feeding system

The sheep were used in the traditional system (harem mating, one litter a year in February–March), and were kept in the alcove and pasture system on deep litter.

During the mating period and early pregnancy (up to the 3rd month), the animals received oat grain (300 g/head/day), lupine (white and narrow-leaved mixture—100 g/head/day) and had ad libitum access to hay. Additionally, in the period of preparation for breeding (from August to the end of September), they had access to pasture. The diet provided to the study herd from the fourth month of pregnancy and during the initial lactation (until the 42nd day post lambing) was based on beet pulp (100 g/head/day), lupine (white and narrow-leaved mixture—600 g/head/day), oat grain (600 g/head/day) and hay ad libitum*.* However, after the 42nd day after giving births, the herd was taken to a pasture with an initial regrowth of 15 cm, which was used for about 8 h a day. During this period, animals received an addition of concentrated feed (oat grain) at the level of 100 g/head/day and had access to hay ad libitum. The grazing period began in April (42 days after giving birth—the difference may reach about 3 days in the study group of sheep). The chemical composition of the feed is presented in Table 6. The feed ration was consistent with the Polish standards—based on INRA method^47^.

**Table 6:** Chemical composition of the fodder^48^.

Sample	Dry matter (%)	Ash (%)	Protein (%)	Fiber (%)	Fat (%)	Gross energy (MJ/kg)
Hay	93.15	7.97	7.54	38.91	1.12	19.91
Oat	95.67	2.22	11.58	11.63	4.3	20.99
Pasture forage	97.03	10.50	6.21	4.12	0.49	21.38
Beet pulp	99.44	9.11	2.34	6.23	0.13	19.08
Lupine mix	88.5	4.6	36.0	8.3	9.2	16.0

### Sample collection and storage

Faecal samples were collected from ewes and their lambs (individually) on the day of lambing (up to 24 h after lambing—day 0, lambs were collected only after nipple expulsion), then on day 28 and 56 after lambing. The faecal saples were collected up to 10 s after defecation (only the top part of the manure, not in contact with the ground) to sterile container, then frozen at − 26 °C until DNA isolation (1 month).

Samples of milk from ewes (on the 0, 28 and 56 days after lambing) were taken without prior cleaning of the teats (to preserve the environment used by the lambs) to sterile container, then they were cooled to 4 °C and frozen. The samples were stored at − 26 °C until the analysis (no longer than 3 months).

### BCS assessment of the studied individuals and lamb's increments

Body condition score (BCS) was carried out for mothers and their lambs at 0, 28, 42 and 56 days after lambing—performed by the same person throughout the research period. It was made on the basis of a 5-point scale according to the methodology described by Thompson and Meyer^49^, wherein:

- 1 point: extreme emaciation.
- 2 points: lean sheep.
- 3 points: sheep of average condition.
- 4 points: hard condition.
- 5 points: obesity.

The evaluation of the growth rate of lambs was carried out by means of control weighing every 2 weeks until the 56th day after lambing (the measurement was performed twice—repeated after about 30 min), and their daily increments were calculated.

### Feed analysis

The collected samples of fodder and pasture forage were subjected to the Wenden analysis (AOAC norm), which was carried out at the Department of Animal Nutrition and Feed Science. The results of the analysis included the determination of the level of dry matter, ash, protein, fibers, NDF (detergent-neutral fiber e.g. cellulose, hemicellulose, lignin), ADF (acidic fiber fractions—least digestible feed components), fat and gross energy^48^.

### Analysis of the composition of ewes' milk

The collected milk samples from day 0, 28 and 56 were analyzed in terms of the content of: fat, protein, lactose, dry matter, and minerals. The analysis was performed using the Infrared Milk Analyzer—150 (Bentley).

### DNA isolation

DNA isolation from feces was performed after prior thawing and mixing of samples (to unify each sample) using Genomic Mini AX Stool (A&A Biotechnology, Gdansk, Poland). DNA from ewe’s milk was isolated from the thawed material using Genomic Mini AX Milk Spin. Both sets were modified by adding mutanolysine and lysozyme (also from A&A Biotechnology, Gdansk, Poland). All the isolations were made in the Laboratory of Microbiology and Molecular Genetics UPWr.

Then, the quality of the isolated DNA was checked using NanoDrop 2000 (Spectrophotometer) from Thermo Scientific. The average DNA content of the samples was 40–50 μg/μl (50 μl) for lamb and 100–150 μl/μg (50 μl) for mother samples. The amount of pollution was at the level of 260/230 (ratio: 2.0–2.2) and 260/280 (ratio: 1.8–2.0).

The main problem in DNA isolation from fecal samples was the occurrence of various inhibitors disturbing the polymerase Taq and/or the primer activity^50^. In case of low quality of the sample obtained, the samples were re-isolated or cleaned of contamination using Clean-up Concentrator (A&A Biotechnology, Gdansk, Poland)^11^.

### Real time analysis

Real-time PCR analysis was done by use of an apparatus BIO-RAD CFX Connect 96 Touch with the help of the kit SsoAdvanced™ Universal SYBR® Green Supermix (Bio-Rad Laboratories, Inc, California, USA) in volume 10 μl in 3 technical repetitions (Table 7), NTC test was additionally performed for each gene. The real-time PCR analysis strategy was based on the amplification of genes specific for the tested phyla against the reference gene for all bacteria. The reference gene was 16S universal Eubacterial gene 530F^51^, whereas for phylum Firmicutes 16S 928F-Firm and 1040FirmR for Bacteroidetes 16S 798cfbF, cfb967R^52^. For family *Lactobacillaceae* were used genes 16S, Lac1 Forward Lac2Seq^53^ (Table 8). For the tested genes a standard curve was made to determine the performance of individual genes. A sample dilution of 10^–6^ from the 10^–2^ to 10^–7^ series of dilutions was selected for analysis. The analysis was performed according to a protocol of 40 cycles: Polymerase Activation and DNA denaturation 95 °C (3 min), denaturation 95 °C (15 s.), annealing 60.5 °C (15 s.), extension and plate read 72.0 °C (40 s). The analysis of the melting curve for the samples at the temperature from 65 °C (5 s) to 95 °C (0.5 °C increments in 2 s).

**Table 7:** Composition of PCR mix.

Component	Volume per 10 μl reaction
SsoAdvanced™ Universal SYBR® Green Supermix	5 μl
Foward and revers primers	1 μl (0.8 μM)
DNA template	2 μl (0.04–0.015 × 10^–4^)
Nuclease—free water	2 μl

**Table 8:** RT-PCR primers.

Name	Forward (5′–3′)	Revers (5′–3′)
Universal eubacterial genes^51^	530F (5′-GTC CCA GCM GCN GCG G)	1100R (5′-GGG TTN CGN TCG TTG)
Firmicutes^52^	928F-Firm (5′-TGA AAC TYA AAG GAA TTG ACG)	1040FirmR (5′-ACC ATG CAC CAC CTG TC)
Bacteroidetes^52^	798cfbF (5′-CRA ACA GGA TTA GAT ACC CT)	cfb967R (5′-GGT AAG GGT TCC TCG CGT AT)
*Lactobacillaceae*^53^	lac1 forward (5′-AGC AGT AGG GAA TCT TCC A)	Lac2Seq (5′-ATTTCACCGCTACACATG)

The data were then compiled using the following software CFX Maestro (Bio-Rad Laboratories, Inc, California)^54^, were one sample with a DNA level of 100 μg/μl and impurities at a level in line with the above mentioned standards, was an arbitrary calibrator. CFX Maestro calculated the results from the number of the reference gene matrix and the differences at the Relative Normalized Expression (ΔΔC_q_) phylum’s level, taking into account the amplification efficiency of individual genes.

### Statistical analysis

The obtained results were analyzed using the Statistica ver. 13.1^55^. The data distribution was checked with the Shapiro–Wilk test. In the case of normal distribution, the calculations were carried out with the use of ANOVA (with repeat measurments), where the influence of the lambing period on: development of lamb body weight, participation of the studied types and families of microorganisms in mothers and their lambs was tested; composition of mothers' milk. In the absence of a normal distribution (sex of lambs), the Kruskal–Wallis test (p ≥ 0.05) was used. The differences for the ANOVA test were determined using the Bonferroni test (p ≥ 0.05). Correlations (Spearman) were also estimated.

### Ethics approval and consent to participate

The authors confirm that the ethical policies of the journal have been adhered to. All animals that qualified for the study were subjected to standard procedures without any harm or discomfort, the milk and fecal samples were collected from the animals at routine works on the farm, and sampling did not require any harmful contact with animals. Therefore the study did not require the consent of the Local Ethical Committee for Animal Experiments (Act of 15 January 2015 on protection animals used for scientific or educational purposes, OJ 2015, 266, implementing the Directive 2010/63/EU of the European Parliament and the Council of 22 September 2010 on the protection of animals used for scientific purposes).

## Data Availability

The datasets generated and/or analyzed during the current study are not publicly available because a part of them belong to a different set of studies (for personal use), but are available from the corresponding author on reasonable request.
